# Synthesis of carbon dot-mediated gold nanostructures: experimental and quantum chemical insights into turn-on fluorescence sensing of *N*-acetylcysteine

**DOI:** 10.3762/bjnano.17.75

**Published:** 2026-08-12

**Authors:** Thuy-Huong Nguyen, Van-Nghia Nguyen, Ha Thi Thu Nguyen, Ha Ngoc Nguyen, Anh Duc Nguyen, Tuyen Viet Nguyen, Truong Xuan Nguyen

**Affiliations:** 1 School of Chemistry and Life Sciences, Hanoi University of Science and Technology, 01 Dai Co Viet, Hanoi, Vietnam; 2 Faculty of Chemistry, Hanoi National University of Education, 136 Xuan Thuy, Hanoi, Vietnam; 3 University of Science and Technology of Hanoi, Vietnam Academy of Science and Technology, 18 Hoang Quoc Viet, Hanoi, Vietnam; 4 University of Science, Vietnam National University, 334 Nguyen Trai, Hanoi, Vietnam

**Keywords:** fluorescence, gold nanoparticles, green synthesis, *N*-acetylcysteine, nitrogen-doped carbon dots

## Abstract

In this work, a green and sustainable hydrothermal approach was employed to synthesize nitrogen-doped carbon dots (NCDs) using lemon juice as a natural precursor. The as-prepared NCDs served as eco-friendly reducing agents and stabilizing scaffolds for the subsequent in situ synthesis of gold nanoparticles (Au@NCDs). The structural and optical properties of the nanocomposite were comprehensively characterized using various analytical techniques. The intrinsic fluorescence of NCDs was significantly quenched upon formation of the composite, mainly due to non-radiative energy transfer processes. Interestingly, the quenched fluorescence could be efficiently restored upon the addition of *N*-acetylcysteine (NAC), enabling the development of an “off-on” fluorescent sensing platform. The sensing mechanism was systematically elucidated through a combination of spectroscopic studies and density functional theory calculations, revealing that fluorescence recovery originates from a ligand exchange process driven by the stronger binding affinity of NAC toward the gold surface, particularly via Au–S interactions. Under optimized conditions, the assay exhibited a robust linear response for NAC concentrations ranging from 4.0 to 20.0 mg/L, with a detection limit of 0.863 mg/L. These results demonstrate that the Au@NCDs system can function as a sensitive and selective OFF–ON fluorescent probe for NAC detection. This study provides a cost-effective and eco-friendly sensing platform with significant potential for analytical applications in pharmaceutical monitoring.

## Introduction

*N*-acetylcysteine (NAC) has been widely employed for over four decades as an antidote in the treatment of paracetamol (acetaminophen) overdose [1–3] and as a mucolytic agent for chronic obstructive pulmonary disease (COPD) [4]. Careful dosage control of NAC is crucial to ensure effective treatment and prevent side effects. Several analytical methods have been conventionally used to determine NAC levels in biological or pharmaceutical samples, including chromatography [5–6] and spectroscopy [7–9]. Currently, electrochemical and optical sensors are gaining prominence in clinical analysis due to their simplicity, requirement for small sample volumes, cost-effectiveness, high specificity, and selectivity [10–16].

Carbon quantum dots (CQDs or CDs) have garnered significant attention due to their strong fluorescence and exhibit significant potential in applications such as photocatalysis, bioimaging, bioanalytical sensing, and pharmaceutical testing [17]. CDs can be synthesized from diverse precursors, including citric acid, carbohydrates, and organic molecules through two distinct methodologies, that are, bottom-up and top-down methods [18–19]. In recent years, carbon dots derived from natural sources have increasingly attracted interest as a sustainable and environmentally friendly approach [20–22]. Natural precursor sources, including plant extracts, fruits, and vegetables (e.g., orange juice [23], turmeric [24], lemon juice [25], mango leaves [26], and guava leaves [27]), are abundant, inexpensive, and renewable carbon sources. Furthermore, they contain various organic compounds with hydroxy, carboxyl, and amino groups. Consequently, natural-product-derived carbon dots frequently demonstrate high hydrophilicity, excellent biocompatibility, and a broader spectrum of surface functional groups compared with CDs synthesised from chemical precursors [28–30].

Among the various strategies aimed at improving the physicochemical properties of CDs, heteroatomic doping has been extensively studied [31]. Nitrogen-doped carbon dots (NCDs) are formed by introducing nitrogen atoms into carbon frameworks or functional groups on the surface. Nitrogen doping alters the electronic structure of the carbon core and creates surface functional groups such as amine and pyridinic groups. Compared with undoped carbon dots, NCDs featured excellent robust fluorescence stability and chemical resistance [32].

Fluorescent probes are powerful analytical tools favored for their rapid response, high sensitivity, simplicity, low cost, and real-time detection capabilities. To overcome the inherent limitations of organic dyes, nanomaterial-based sensing platforms have attracted considerable research interest. In particular, gold-based composite nanoparticles stand out as promising candidates for advanced fluorescent probes, owing to their exceptional optical properties, large specific surface area, excellent biocompatibility, and ease of surface functionalization [33]. A key feature of gold nanoparticles is their surface plasmon resonance, which generates strong light–matter interactions, amplifies optical signals, and underpins their extensive application in optical sensing [34].

This study developed an OFF–ON fluorescent sensor based on gold nanoparticles and nitrogen-doped carbon dots (Au@NCDs) for the determination of NAC. NCDs, possessing strong luminescence properties, served as both reducing agent and stabilizing agent for the synthesis of Au@NCDs [35]. Upon excitation of the NCDs, the energy was transferred to the gold nanoparticles, resulting in fluorescent quenching. With the addition of NAC, a ligand exchange occurred between the thiol groups of NAC and the NCDs on the gold nanoparticles surface, resulting in the formation of a stronger Au–S bond and the subsequent recovery of the fluorescent signal. The restored fluorescence intensity exhibited a proportional relationship with the concentration of the added NAC.

In addition to experimental characterization, density functional theory (DFT) calculations have emerged as a powerful approach for elucidating the molecular-level mechanisms governing the performance of nanomaterial-based fluorescent sensors [36–38]. DFT provides valuable insights into adsorption configurations, binding energies, charge redistribution, and electronic structures, thereby enabling the interpretation of analyte–nanomaterial interactions that are often difficult to resolve experimentally. In particular, for gold nanostructure-based sensing platforms, DFT has been widely employed to investigate the formation and stability of Au–S bonds [39–40], ligand exchange processes [41–42], and the associated electronic changes responsible for fluorescence quenching and recovery [43–45]. Therefore, integrating DFT calculations with experimental studies offers a comprehensive understanding of the sensing mechanism and facilitates the rational design of highly sensitive fluorescent nanosensors.

Although carbon dots and gold nanoparticles have been widely investigated for fluorescence sensing, the development of lime juice-synthesized carbon dot-mediated gold nanostructures with mechanistic insight into their fluorescence response toward NAC remains limited. In particular, the relationship between the structural and electronic properties of Au@NCD nanocomposites and their fluorescence sensing behavior has not been fully understood. Therefore, the novelty of this study lies not only in using a natural source for synthesis of Au@NCD nanostructures but also in the combination of experimental and theoretical approaches to provide mechanistic insights into the fluorescence sensing process.

## Experimental

### Chemicals and instrumentation

Tetrachloroauric(III) acid trihydrate (99.5%), urea (99.5%), dimethylformamide (DMF, ≥99.8%), acetone (≥99.8%), and silicagel 60 (0.015–0.040 mm) were purchased from Merck, USA. *N*-Acetylcysteine ≥98% and ethanol 95% were supplied by ThermoFisher, USA. Acetonitrile (ACN, ≥99.9%) was bought from Fisher Scientific, England. Fresh lime juice was prepared from limes (*Citrus × aurantiifolia*) obtained from a local supermarket in Hanoi, Vietnam. The chemicals were used as received, and their solutions were prepared in double distilled water. Fluorescence measurements were carried out using a Jasco FP-6500 spectrofluorometer (Japan). Absorption spectra were collected on an Agilent 8453 UV–vis spectrophotometer (USA). Fourier-transform infrared (FTIR) spectra were recorded by a Nicolet iS50 spectrometer from Thermo Scientific (Japan). Morphologies and sizes of the products were characterized using a JEOL JEM-1010 Transmission electron microscope (TEM), and a JEOL JSM-IT800 field emission scanning electron microscope (FESEM) (Japan). The elemental analysis was carried out on an Oxford EDX Ultim Max 65 (England) coupled with the FESEM instrument. Raman spectra of the samples were taken with an acquisition time of 60 s in two cycles by using HR 800 spectrometer from Horiba Jobin Yvon (France). The size distribution was characterized using a dynamic light scattering (DLS) Instrument (Horiba SZ-100-Z2, Japan). The glass chromatographic column with 45 cm in length and 2 cm in diameter was purchased from Duran, Germany.

### Synthesis of nitrogen-doped carbon dots

NCDs were synthesized via a modified hydrothermal method [46]. Typically, a mixture of 20 mL of fresh lemon juice and 0.8 g of urea was stirred, transferred into a 50 mL Teflon-lined autoclave, and heated at 180 °C for 3 h. Afterward, the autoclave was allowed to cool naturally to room temperature. The resulting mixture was purified by gradient silica gel column chromatography, using approximately 100 mL acetone followed by 250 mL acetone/distilled water mixture (4:1, v/v) as the mobile phase. The purified CD fractions were identified by collecting sequential eluates and monitoring their fluorescence under UV light (365 nm) and PL spectroscopy. Fractions exhibiting an intense characteristic fluorescence of CDs were combined, while non-fluorescent or weakly fluorescent fractions were discarded [47].

### Preparation of Au@NCDs composite

The synthesis conditions of Au@NCDs composites were primarily optimized including NCDs/HAuCl_4_ ratio, temperature, and reaction time (see section 2 in Supporting Information File 1). The Au@NCDs composite was obtained by adding 2.5 mL of a NCD solution (0.15 mg/mL) to 2.5 mL of 0.2 mM HAuCl_4_ under continuous stirring and heating at 60 °C. After 5 min, the solution gradually changed in color from yellow to pink. The mixture was then left at room temperature for 15 min before use [16,48]. The resulting mixture was centrifuged at 10,000 rpm for 30 min, and the supernatant was discarded. The precipitate was redispersed in deionized water (DW) and stored for further use.

### Optimization of the organic solvent composition

The effect of the organic solvent on the fluorescence response of the Au@NCDs sensing system was investigated using mixtures of aqueous and organic solvents. Methanol, ethanol, acetone, ACN, and DMF were individually mixed with DW at a volume ratio of 25:75 (v/v). NAC standard solutions (25 mg/L) were prepared in each solvent mixture under identical conditions.

For each experiment, an aliquot of the Au@NCDs probe was mixed with the corresponding NAC solution, and the fluorescence intensity was recorded after incubation under the optimized sensing conditions described in section “Preparation of Au@NCDs composite”. The fluorescence recovery was evaluated as the change in fluorescence intensity (Δ*I*), calculated from the difference between the fluorescence intensity of the Au@NCDs probe in the presence (*I*) and absence (*I*_0_) of NAC (Δ*I* = *I* − *I*_0_). All measurements were performed in triplicate, and the average values were used for comparison.

After identifying DMF as the most suitable organic solvent, its proportion in the water/DMF mixture was further optimized. Water/DMF mixtures containing 5%, 10%, 15%, 20%, and 25% (v/v) DMF were prepared while maintaining the NAC concentration at 25 mg/L. Fluorescence measurements were carried out under identical experimental conditions, and the corresponding fluorescence recovery (Δ*I*) was compared to determine the optimal solvent composition. Based on these experiments, a water/DMF mixture containing 25% (v/v) DMF was selected and used for all subsequent fluorescence sensing experiments.

### Assay of *N*-acetylcysteine

Fresh standard NAC solutions were prepared prior to analysis and only stable Au@NCDs dispersions prepared using the same synthesis protocol were used for measurements. A mixture of DW and DMF solvent was used as a blank to zero background signals. All fluorescence measurements were performed under identical instrumental conditions and each experiment was conducted in triplicate. A specific amount of DMF solvent was added to 1 mL of Au@CDs solution, followed by 0.5 mL of NAC standard solution at concentration ranging from 1.0 to 80.0 mg/L. The resulting mixture was then equilibrated for 5 min. Subsequently, the fluorescent spectra of the prepared solutions were recorded. All measurements were performed in triplicate, and the average values were used for calculation. The analytical linear range and limit of detection were calculated from the obtained data.

### Computational details

All calculations were carried out within the framework of DFT using the SIESTA package [49]. The exchange–correlation energy was described by the generalized gradient approximation with the Perdew–Burke–Ernzerhof functional [50]. Dispersion interactions were included using the Grimme DFT-D3 dispersion correction scheme with default parameters [51–52]. Norm-conserving pseudopotentials were used to represent the ionic cores, and valence states were expanded in a double-ζ plus polarization (DZP) numerical atomic orbital basis set for all atoms. The real-space integration grid was defined by a mesh cutoff of 4761.99 eV, which was verified to ensure convergence of total energies. Electronic occupations were treated using a Fermi–Dirac distribution at 300 K. Self-consistent field calculations were performed using the diagonalization method with Pulay mixing (mixing weight 0.15). Spin-polarized calculations were employed throughout the study.

**Figure 1:**
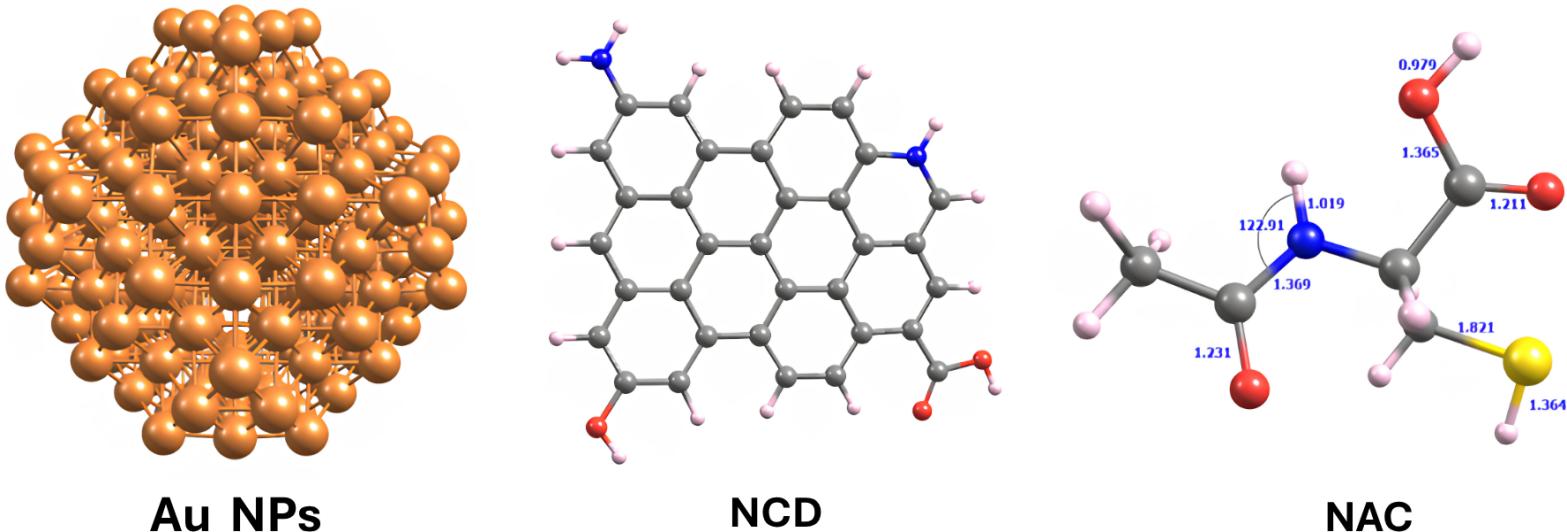
Optimized structures of (a) Au NPs, (b) NCD, (c) NAC. Color codes: light brown – Au, grey – C, blue – N, red – O, yellow – S, ivory – H. All key distances are in angstroms, angles are in degrees.

The simulated system consisted of a gold nanocluster containing 249 Au atoms, which serves as a representative model for metallic Au nanoparticles (Au NPs) [53], interacting with a NCD model and NAC (Figure 1). The optimized structure of NAC shows good agreement with experimental data, with calculated geometric parameters deviating from reported values by approximately 1.17–3.4% (see Figure S2 and Table S2 in Supporting Information File 1), confirming the reliability of the computational approach. The NCD structure was constructed based on reported structural models, incorporating surface functional groups such as –NH_2_, –OH, and –COOH. The model consists of 54 atoms, with edge carbon atoms hydrogen-passivated to satisfy valence requirements. All systems were modeled under periodic boundary conditions using sufficiently large simulation cells to avoid interactions between periodic images. Owing to the presence of acidic functional groups (–COOH, –SH), the interactions between NAC/NCD and Au NPs were systematically investigated for both neutral species (NAC and NCD) and their corresponding deprotonated forms (NAC^−^ and NCD^−^). This approach allowed for a comprehensive evaluation of binding behavior under different chemical environments and provides a basis for understanding the ligand exchange mechanism discussed in subsequent sections.

## Results and Discussion

### Structural and morphological analysis

**Figure 2:**
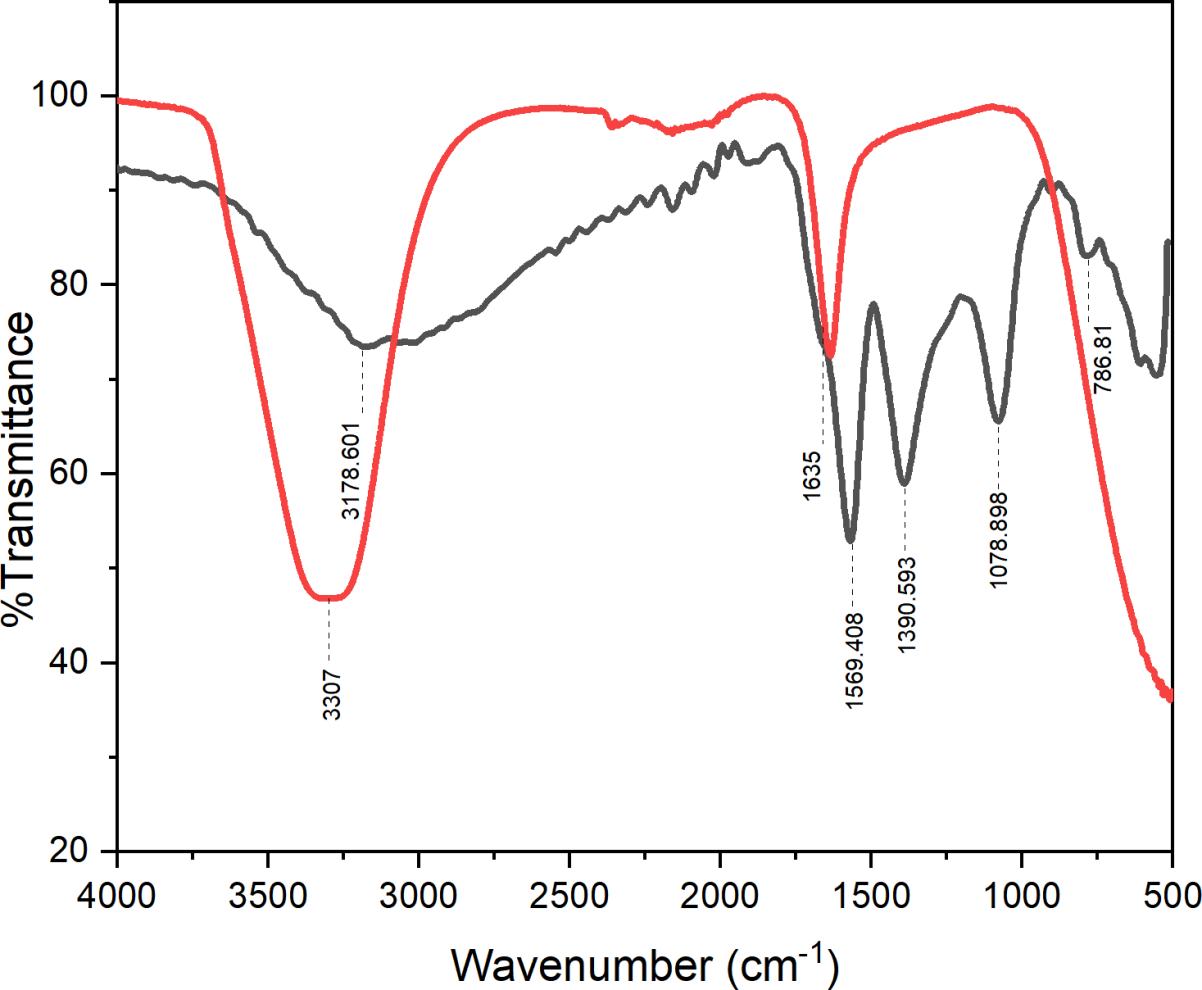
FTIR spectra of NCDs (black line) and Au@NCDs (red line).

Characteristic functional groups of NCDs such as hydroxy, amine, carboxyl and carbonyl groups [54–56] are evident in the FTIR spectrum in Figure 2. Specifically, the broad absorption band in the 3000 to 3500 cm^−1^ region can be attributed to the stretching vibration of –NH/–OH group (υ_N–H_/υ_O–H_). Absorption peaks around 1635 cm^−1^ (shoulder) and 1569 cm^−1^ are assigned to υ_C=O_, while peaks at 1390 cm^−1^ correspond to C–N stretching vibrations. Vibration of –CO/CN/CH groups are responsible for the absorption peaks around 1078 cm^−1^. The C–H stretching vibrations of benzene are represented by the peak at 786 cm^−1^.

**Figure 3:**
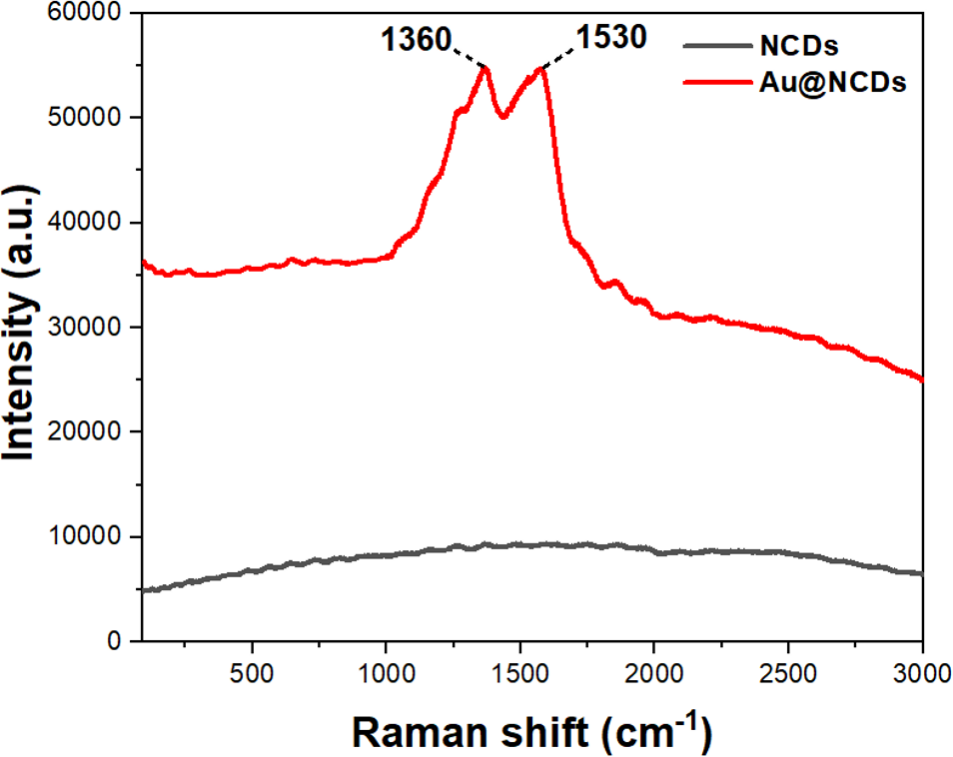
Raman spectra of NCDs and Au@NCDs.

Figure 3 shows the Raman spectra of NCDs and Au@NCDs. The NCD spectrum lacked discernible peaks due to significant fluorescence interference while Au@NCDs clearly shows the D band and the G band of carbon at 1360 and 1530 cm^−1^, respectively [17]. Fluorescence quenching of carbon dots by Au nanostructures is a well-documented phenomenon where the fluorescence intensity of CDs decreases upon the addition of Au^3+^. The quenching of the NCDs’ photoluminescence can occur due to different mechanisms. The quenching can be attributed to a redox reaction where the Au^3+^ ions interact with the surface functional groups of the CDs, leading to a redox reaction, disrupting the normal radiative decay of the excited state in the CDs and thus reducing fluorescence [57]. Another mechanism for quenching is aggregation [58]. Reaction with Au^3+^ can also induce aggregation of the CDs, leading to a reduction of surface area and increased non-radiative decay pathways, while suppressing the fluorescence process. Moreover, the fluorescence of CDs might be quenched through fluorescence resonance energy transfer (FRET), a process involving energy transfer from the CD donor to the AuNP acceptor [59–60].

**Figure 4:**
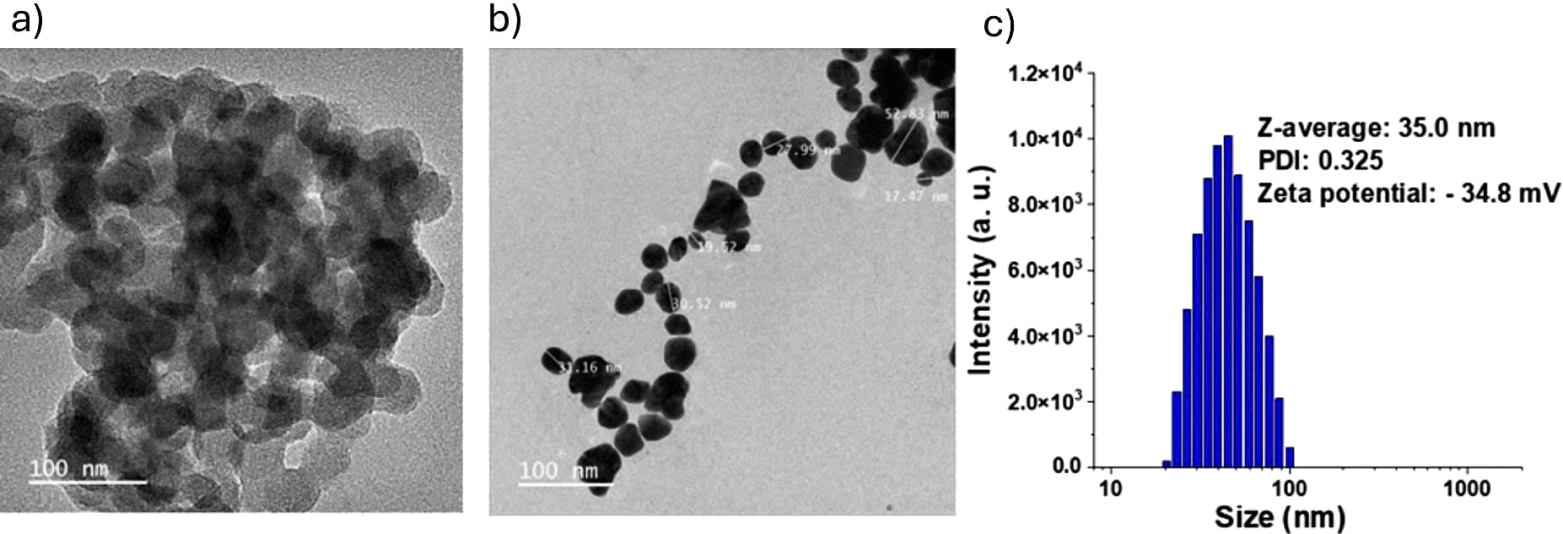
TEM images of (a) NCDs and (b) Au@NCDs and (c) DLS size distribution analysis of Au@NCDs.

As can be seen in Figure 4a, NCDs tend to form clusters with an average size of 30 nm size. In contrast, a mixture of spherical, polyhedral, triangular, and rod-like Au nanostructures is observed with Au@NCDs (Figure 4b). The diameter of Au NPs was approximately 35 nm, which is in agreement with dynamic laser scattering (DLS) analysis (Figure 4c). Indeed, the DLS results showed that the Au@NCD nanoparticles have sizes from 20 to 90 nm, while the average value is 35 ± 16.4 nm. The recorded PDI of 0.35 for the Au@NCDs indicated a highly polydispersed system resulting from a mixture of different structural Au nanoparticles. In addition, the zeta potential of −34.8 mV indicated a stable colloidal solution of Au@NCDs in water.

**Figure 5:**
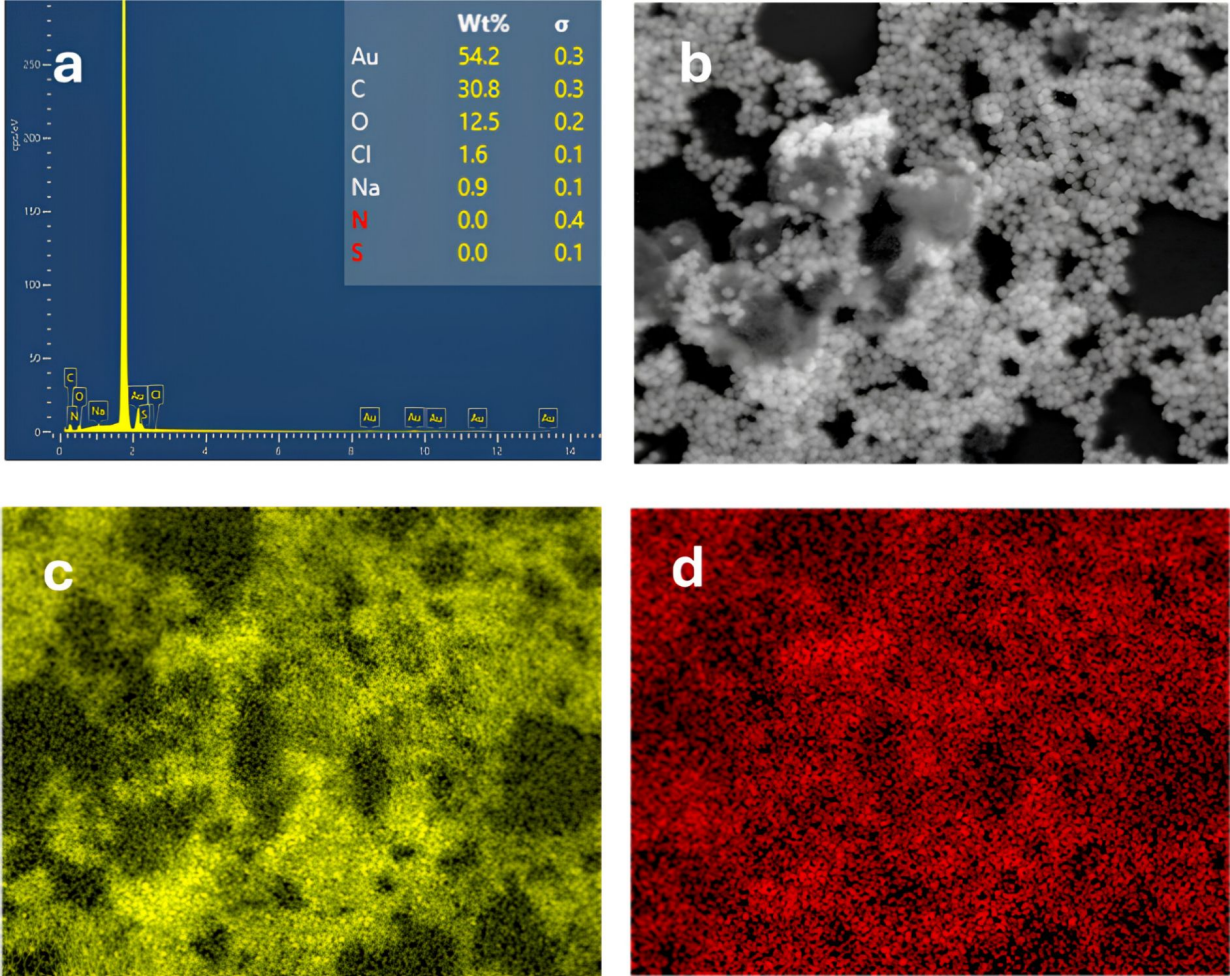
(a) EDX spectrum with elemental weight percentages, (b) SEM image, and elemental mapping of (c) Au and (d) C in Au@NCDs.

**Figure 6:**
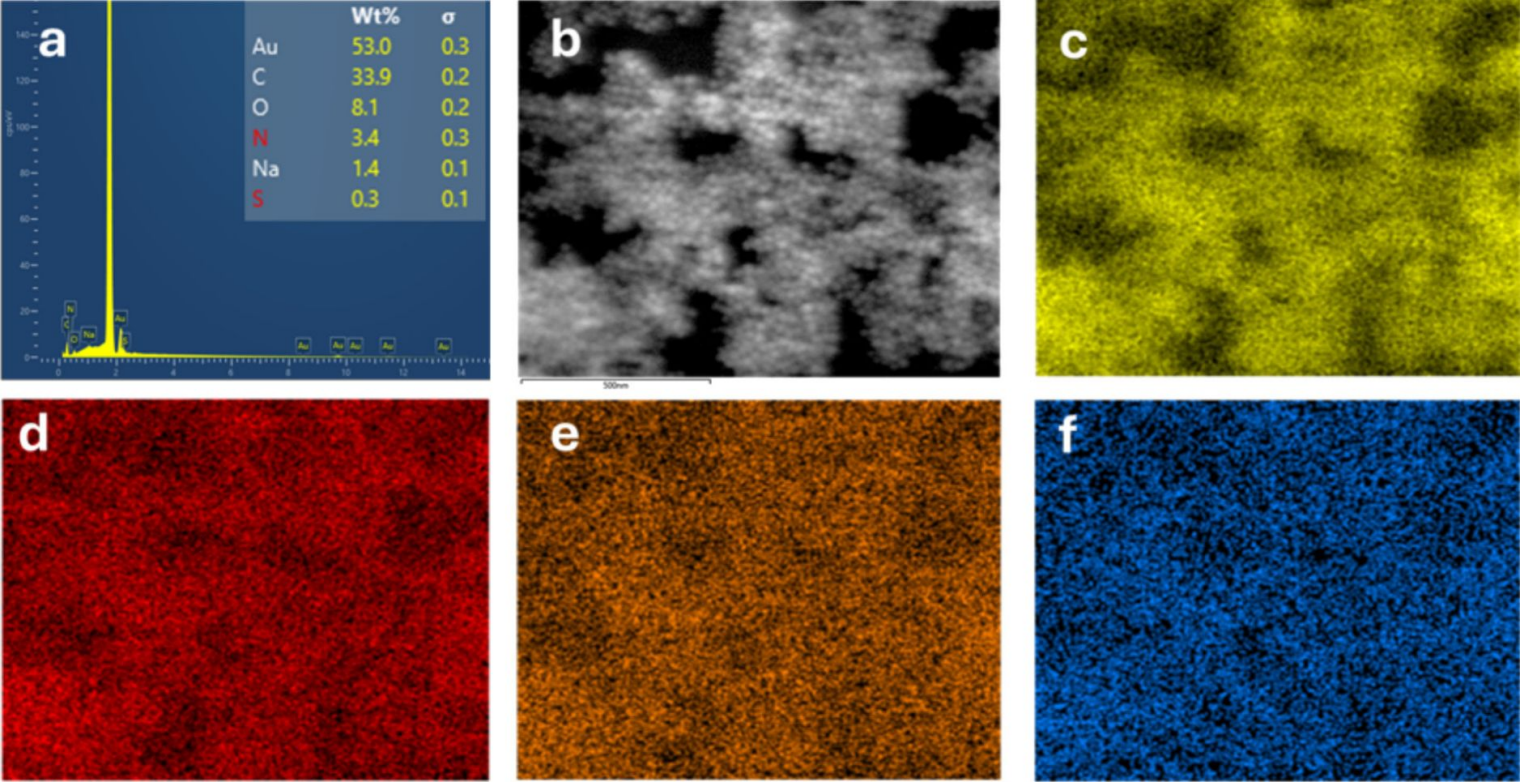
(a) EDX spectrum with elemental weight percentages, (b) SEM image, and elemental mapping of (c) Au, (d) C, (e) S, and (f) N in Au@NCDs-NAC.

The element distribution on Au@NCDs and Au@NCDs-NAC were analyzed by FESEM in combination with energy dispersed X-ray spectroscopy as shown in Figure 5 and Figure 6, respectively. The spectra indicated that there were Au and C in Au@NCDs and Au, C, N, and S in Au@NCDs-NAC. The Au and C mapping images revealed the strong interaction between Au nanoparticles and NCDs in all samples. The presence of S in the EDX spectra of Au@NCDs-NAC suggested that the NAC molecules have interacted closely with NCDs.

### Photophysical properties

**Figure 7:**
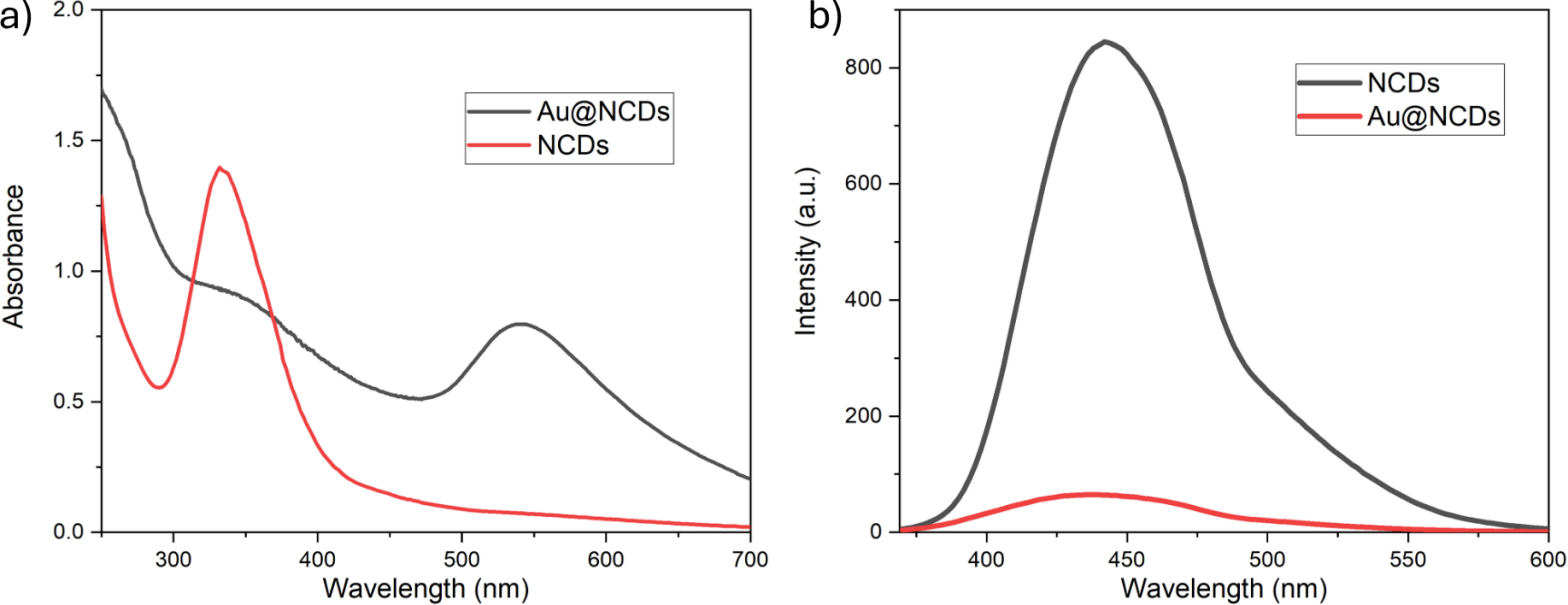
(a) Absorption and (b) photoluminescence spectra of NCDs and Au@NCDs (λ_ex_ = 355 nm).

Figure 7a shows the absorption spectra of NCDs and Au@NCDs. A distinct peak at 340 nm can be assigned to the π→π* transition of carbonyl C=O bonds present on the surface of NCDs. In the absorption spectrum of the Au@NCDs composite, this characteristic peak vanished due to the strong electronic interactions between the gold nanoparticles and NCDs [16]. Furthermore, the formation of Au NPs is confirmed by the presence of a broad surface plasmon resonance band centered around 550 nm.

Figure 7b presents photoluminescence (PL) spectra of NCDs and Au@NCDs when excited at 355 nm. It can be seen clearly that the Au nanoparticles quenched the fluorescence of NCDs, which is in agreement with the Raman data. Fluorescence quenching of NCDs can be attributed to several factors. The fluorescence of NCDs might be quenched through FRET. Such mechanism is reasonable as evidence by the spectral overlap of NCDs emission and Au@NCDs absorption.

### Application of Au@NCDs as a fluorescent probe for detection of *N*-acetylcysteine

#### Sensing mechanism

**Figure 8:**
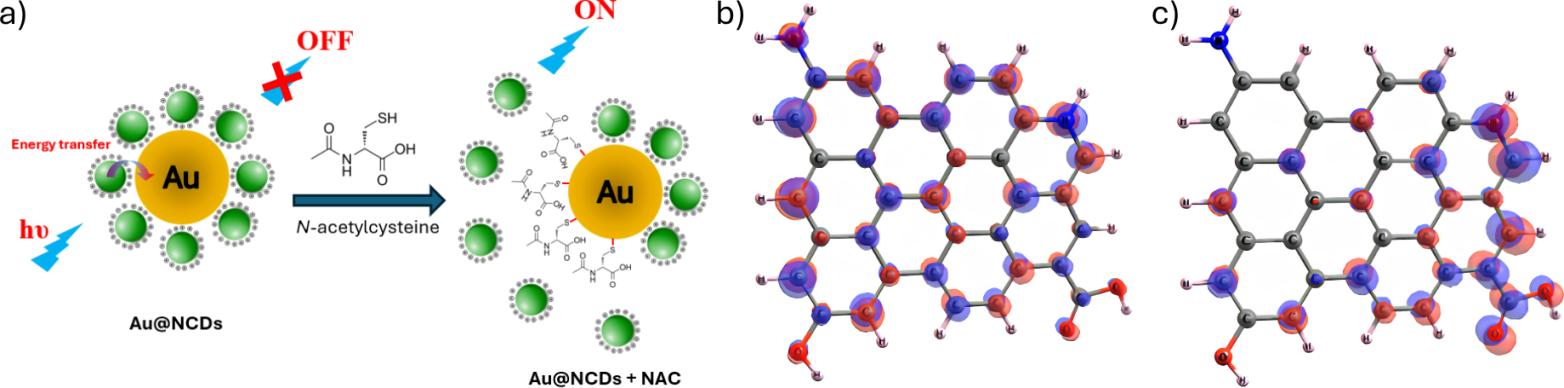
(a) Schematic illustration of the NAC detection mechanism of the Au@NCDs probe, (b, c) spatial distributions of the HOMO and LUMO of NCDs.

The mechanism of NAC detection by the Au@NCDs probe is schematically illustrated in Figure 8a. In this system, NCDs act as light-harvesting antennae, efficiently absorbing incident photons and generating excited electronic states. The calculated frontier orbital energies of NCDs show that the HOMO and LUMO are located at −8.5746 and −8.0600 eV, respectively, corresponding to a relatively small energy gap (≈0.51 eV), which facilitates photoexcitation under mild conditions.

The spatial distribution of the frontier orbitals further reveals that the HOMO is mainly localized on the π‑conjugated carbon framework, while the LUMO is more delocalized and extends toward surface functional groups containing heteroatoms such as oxygen and nitrogen, consistent with previous reports on heteroatom‑doped carbon dots (Figure 8b,c). This redistribution of electron density upon excitation suggests a partial intramolecular charge transfer character, enhancing the photoactive nature of NCDs and supporting their role as effective energy donors [61]. Rather than undergoing direct electron transfer, the excitation energy is transferred to Au nanoparticles via a non-radiative energy transfer process, likely mediated by plasmonic coupling or dipole–metal interactions. Notably, Au NPs exhibit a near-zero HOMO–LUMO gap, reflecting their metallic electronic structure and quasi-continuous density of states, which renders a discrete frontier orbital description inappropriate. Therefore, Au NPs function as efficient energy acceptors, where the transferred excitation energy is rapidly dissipated through non-radiative relaxation pathways, leading to fluorescence quenching (OFF state).

Upon the introduction of NAC, a ligand exchange process is proposed to occur, in which NAC molecules displace NCDs from the Au NPs surface. This behavior can be attributed to the stronger binding affinity of NAC toward Au. This proposed mechanism is further supported and rationalized by quantum chemical calculations. The computed interaction energies for Au NPs interacting with NCD and NAC provide molecular-level insight into their relative binding strengths, confirming the preferential adsorption of NAC on the Au NPs surface. These results offer a deeper understanding of the microscopic origin of the ligand exchange process and its role in modulating the fluorescence response of the Au@NCDs probe. The interaction energy *E*_int_ is defined as:


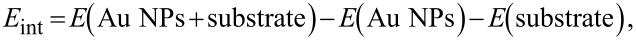


where (*E*(Au NPs + substrate), *E*(Au NPs), *E*(substrate) denote the total energies of the Au NPs bound to the substrate (NCD, NAC, NCD^−^, or NAC^−^), the isolated Au NPs, and the isolated substrate molecules (NCD, NAC, NCD^−^, or NAC^−^), respectively.

**Figure 9:**
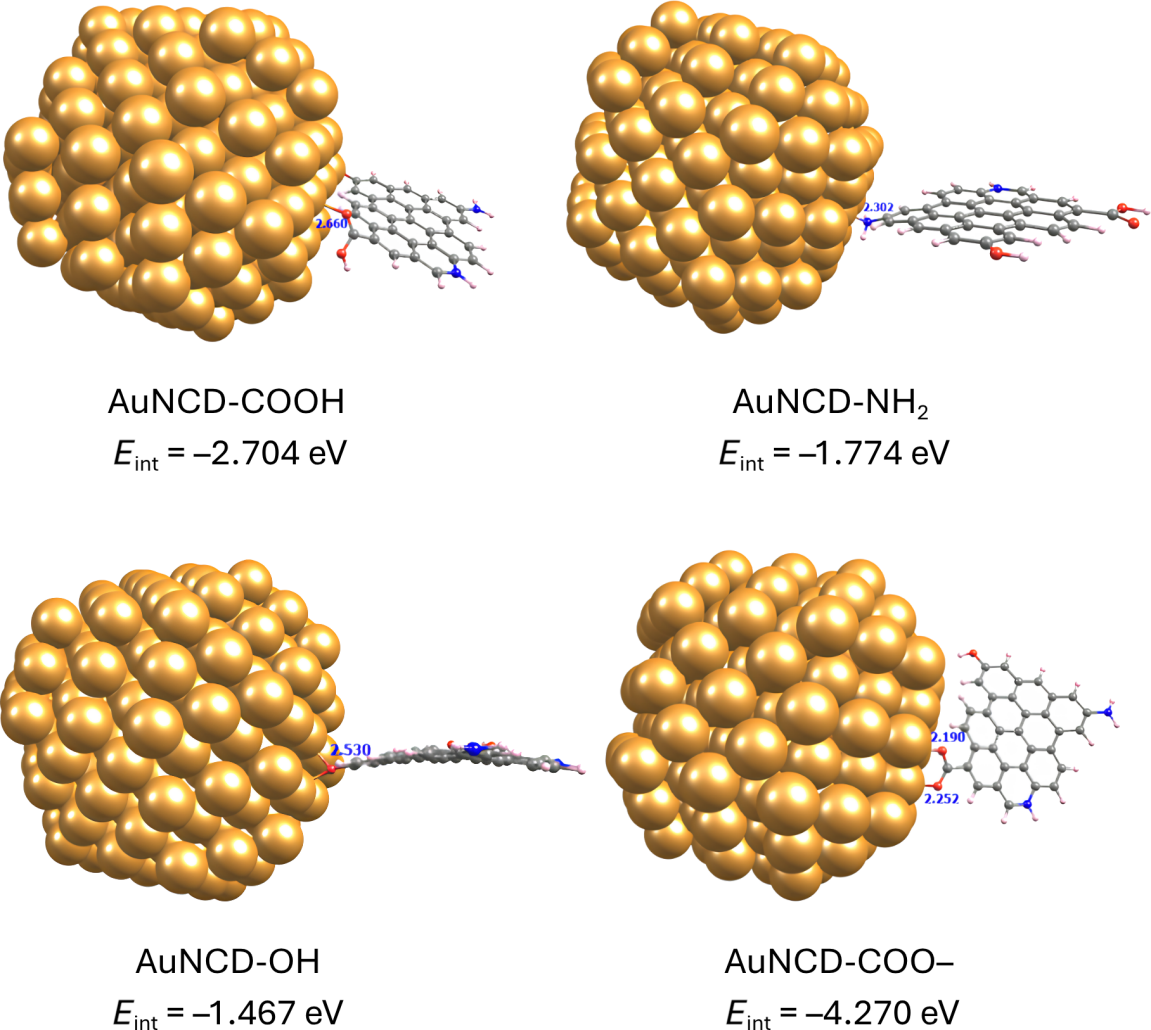
Optimized structures of the binding modes between Au NPs and NCD, along with the corresponding interaction energies.

First, the interaction between Au NPs and NCD was evaluated. Owing to the presence of several functional groups on its surface, Au NPs can interact with NCD at multiple sites, namely, via the –COOH group (forming AuNCD–COOH), via the –NH_2_ group (forming AuNCD–NH_2_), via the –OH group (forming AuNCD–OH), and through interaction with the deprotonated NCD^−^ species, assuming proton dissociation from the –COOH group to form AuNCD–COO^−^. The optimized structures along with the corresponding *E*_int_ are presented in Figure 9.

Among the considered configurations, the deprotonated carboxyl group (–COO^−^) exhibits the strongest interaction with the Au surface, with an *E*_int_ of −4.270 eV, which can be attributed to enhanced electrostatic attraction and strong Au–O coordination. The relatively short interaction distances (2.19–2.25 Å) further support the formation of stable binding in this configuration. In comparison, the neutral –COOH and –NH_2_ groups show moderate interaction strengths, suggesting coordination interactions through lone pair donation from O or N atoms to the Au surface. The –OH group exhibits the weakest interaction (*E*_int_ = −1.467 eV). These results demonstrate that NCDs can bind to Au NPs through multiple functional groups with varying affinities, depending on their chemical state. Importantly, this moderate and tunable binding suggests that the NCD layer on Au NPs may be susceptible to displacement by ligands with stronger binding affinity, which will be further discussed in comparison with NAC.

**Figure 10:**
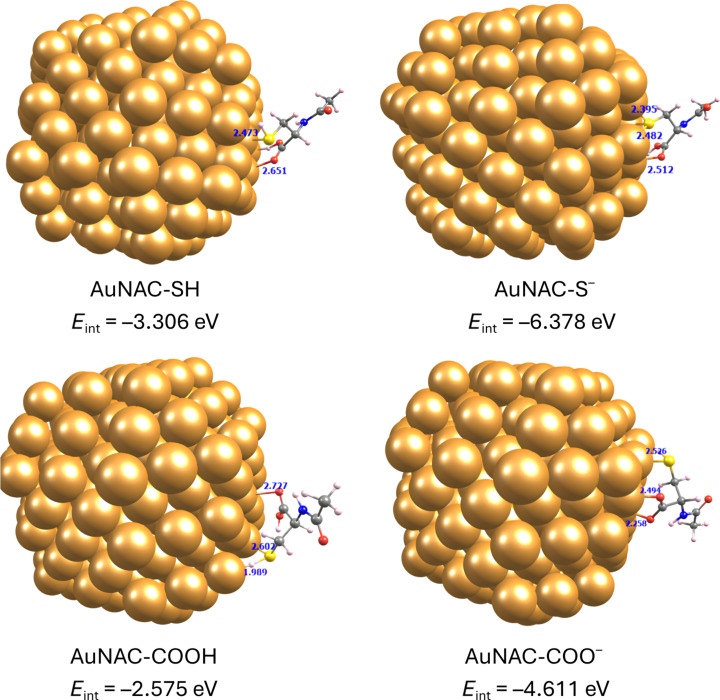
Optimized structures of the binding modes between Au NPs and NAC, along with the corresponding interaction energies.

Building upon the analysis of Au NPs and NCD interactions, it is essential to further examine the binding behavior of NAC on the Au surface in order to understand the ligand exchange mechanism. The optimized structures, together with their corresponding interaction energies (Figure 10), provide detailed insight into the binding characteristics of NAC on the Au NPs surface.

**Figure 11:**
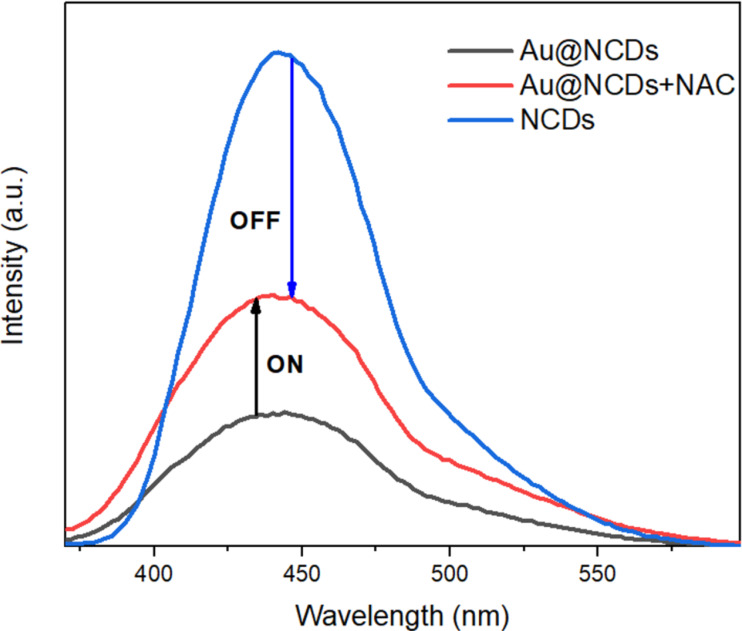
Photoluminescence spectra of NCDs, Au@NCDs, and Au@NCDs after adding NAC.

Among these configurations, the thiol group (–SH) plays a dominant role in anchoring NAC onto Au NPs. Even when the –COOH group is initially oriented toward the surface, structural optimization consistently leads to a configuration in which NAC binds through the thiol group, with an interaction energy of approximately −2.575 eV. This value is weaker than the configuration in which NAC simultaneously interacts with the Au surface via both the –SH and –COOH groups, which yields a stronger interaction energy of −3.306 eV. It is noted that, the deprotonated thiolate form (–S^−^) exhibits significantly stronger binding, with an interaction energy of −6.378 eV, due to enhanced electron donation and stronger Au–S coordination, often accompanied by multipoint bonding interactions with the Au surface. These results clearly indicate that NAC interacts predominantly and most strongly with Au NPs through the sulfur head group. Importantly, when compared with the NCD system, NAC consistently exhibits more negative interaction energies, indicating a stronger binding affinity toward Au NPs. This difference provides a thermodynamic driving force for the ligand exchange process, in which NAC molecules effectively displace NCDs from the Au surface. As a consequence, the displacement of NCDs disrupts the energy transfer pathway between NCDs and Au NPs, thereby restoring the fluorescence signal (ON state) of the sensing system. These results clearly indicate that NAC interacts predominantly and most strongly with Au NPs through the sulfur head group. Importantly, when compared with the NCD system, NAC consistently exhibits more negative interaction energies, indicating a stronger binding affinity toward Au NPs. This difference provides a thermodynamic driving force for the ligand exchange process, in which NAC molecules effectively displace NCDs from the Au surface. The stronger adsorption of NAC increases the separation between NCDs and Au nanoparticles, thereby disrupting the non-radiative energy transfer pathway responsible for fluorescence quenching. Because this energy transfer is highly dependent on the close proximity between NCDs and Au nanoparticles, weakening their interaction suppresses the quenching process and restores the fluorescence emission of NCDs, giving rise to the characteristic OFF–ON fluorescence response. This mechanistic interpretation establishes a direct correlation between the DFT-calculated interaction energies and the experimental photoluminescence results. The proposed ligand exchange mechanism and the associated fluorescence recovery are further supported by experimental evidence from PL spectra (Figure 11).

As shown, pristine NCDs exhibit strong fluorescence emission, whereas the emission intensity is significantly quenched upon formation of Au@NCDs, confirming the effective non-radiative energy transfer from NCDs to Au NPs (OFF state). Upon the introduction of NAC, the fluorescence intensity is partially restored, indicating disruption of the NCD–Au interaction and suppression of the energy transfer pathway. This recovery (ON state) is consistent with the displacement of NCDs from the Au surface by NAC molecules, as suggested by the stronger binding affinity of NAC revealed in the theoretical calculations. Furthermore, previous studies have reported that gold nanocomposites exhibit good selectivity toward NAC, suggesting that the proposed CD@Au nanocomposite is also likely to be selective for NAC [11].

Additional evidence supporting the proposed sensing mechanism can be obtained from Raman spectroscopy (Figure S3 in Supporting Information File 1). In the presence of NAC, the Raman intensity corresponding to the C–H bending (δ_CH_) vibrations decreases, which can be attributed to the recovery of NCD fluorescence. This behavior is consistent with the fluorescence–Raman interplay, where enhanced fluorescence emission can partially suppress Raman signals. Taken together, both the PL and Raman results are in good agreement with the theoretical findings, providing complementary evidence for the ligand exchange-driven sensing mechanism and the associated OFF–ON fluorescence response of the Au@NCDs probe.

#### Effect of organic solvent

**Figure 12:**
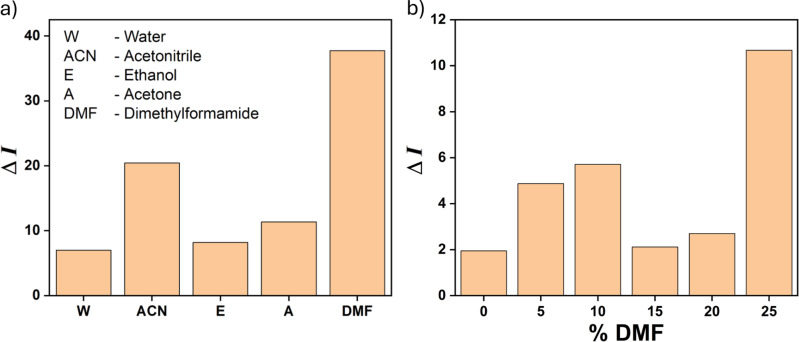
(a) Effect of organic solvent and (b) amount of DMF solvent ratio.

Figure 12a illustrates the fluorescence response of the Au@NCDs probe in a 25 mg/L NAC solution comprising water and an organic solvent (75:25 v/v). The results indicated the ON fluorescence intensity varied with the solvent environment. The fluorescence response is likely influenced by specific solvation and solvent–surface interactions. Among the tested solvents, the highest fluorescence recovery was observed in the presence of DMF. This behavior was consistently demonstrated through fluorescence measurements. To minimize the organic solvent usage in the assay, the DMF ratio was investigated across a range of 5% to 25%. As depicted in Figure 12b, a reduction in the DMF percentage led to a diminished recovered fluorescence intensity. Consequently, a DMF ratio of 25% was maintained constant throughout the study.

### Spectrofluorimetric determination of *N*-acetylcysteine

**Figure 13:**
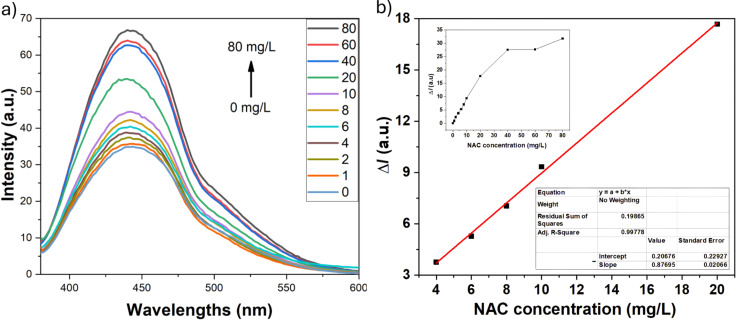
(a) The fluorescence spectra of Au@NCDs + NAC (0–80 mg/L); (b) calibration curve.

As shown in Figure 13a, the fluorescence intensity of the Au@NCDs probe increased progressively with increasing NAC concentration. A good linear relationship was obtained over the concentration range of 4.0–20.0 mg/L (Figure 13b), which is described by the regression equation:


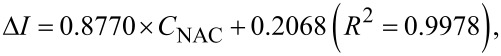


where Δ*I* is the fluorescence intensity change and *C*_NAC_ is the NAC concentration (mg/L). At concentrations higher than 20.0 mg/L, the fluorescence recovery gradually approached saturation, resulting in a reduced analytical sensitivity.

The limit of detection (LOD) and limit of quantitation (LOQ) of the proposed assay were determined based on the validated calibration range, according to the following equations:


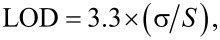



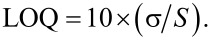


Here, *S* is the slope of the linear calibration curve, and σ represents the standard deviation of the response (Δ*I*). The estimated LOD and LOQ were 0.863 mg/L and 2.615 mg/L, respectively. Since the validated calibration range of the proposed method extends from 4.0 to 20.0 mg/L, the calculated LOD and LOQ should be regarded as statistical estimates of the detection and quantification capabilities based on the standard response. Reliable quantitative determination is therefore supported within the validated calibration range, while extension of the calibration curve to lower concentrations would be required to experimentally verify the practical lower limit of quantification. The analytical performance of the proposed assay is comparable to that of previously reported spectrofluorimetric methods for NAC determination [11]. Nevertheless, additional matrix-spike experiments are required for complete method validation and should be included in future studies.

## Conclusion

NCDs were successfully synthesized via a facile green process based on modified hydrothermal method from fresh lemon juice and urea as precursors. These NCDs function as both reducing and stabilizing agents in the subsequence synthesis of Au@NCDs. The results demonstrate the OFF–ON fluorescence behavior of the Au@NCDs in the absence and presence of *N*-acetylcysteine, a phenomenon attributed to a combination of energy transfer and ligand exchange. This proposed method holds potential for the quantitative determination of *N*-acetylcysteine.

## Supporting Information

File 1 Additional information.

## Data Availability

All data that supports the findings of this study is available in the published article and/or the supporting information of this article.
