# Supplementary material for: Synthesis of carbon dot-mediated gold nanostructures: experimental and quantum chemical insights into turn-on fluorescence sensing of N-acetylcysteine
Source: Beilstein J Nanotechnol. 2026 Aug 12;17:1103–16. doi: 10.3762/bjnano.17.75 (PMC13478897; doi:10.3762/bjnano.17.75)
Supplement: Supplementary file 1 [file Beilstein_J_Nanotechnol-17-1103-s001.pdf]

BEILSTEIN JOURNAL OF NANOTECHNOLOGY

# Supporting Information

for

# Synthesis of carbon dot-mediated gold nanostructures: experimental and quantum chemical insights into turn-on fluorescence sensing of *N*-acetylcysteine

Thuy-Huong Nguyen, Van-Nghia Nguyen, Ha Thi Thu Nguyen, Ha Ngoc Nguyen, Anh Duc Nguyen, Tuyen Viet Nguyen and Truong Xuan Nguyen

*Beilstein J. Nanotechnol.* **2026,** *17,* 1103–1116. doi:10.3762/bjnano.17.75

# Additional information

License and Terms: This is a supporting information file under the terms of the Creative Commons Attribution License (https://creativecommons.org/licenses/by/4.0). Please note that the reuse, redistribution and reproduction in particular requires that the author(s) and source are credited and that individual graphics may be subject to special legal provisions.
The license is subject to the *Beilstein Journal of Nanotechnology* terms and conditions: (https://www.beilstein-journals.org/bjnano/terms)

**Table of Contents**


1. Experimental data S2
1.1. General considerations S2
1.2. Preparation of Au@NCDs composite S2
2. Computational details S3
3. Photophysical measurements S4
4. Analytical performance comparison S6
3. References S8


## 1. Experimental data

### 1.1 General considerations

Tetra chloroauric (III) acid trihydrate, 99.5%; urea, 99.5%; dimethylformamide (DMF), ≥99.8%, acetone, ≥99.8% and silicagel 60 (0.015 – 0.040 mm) were purchased from Merck, USA. N-acetyl cysteine ≥98% and ethanol 95% were supplied by ThermoFisher, USA. Acetonitrile (ACN) ≥99.9% was bought from Fisher Scientific, England. Fresh lemon juice was prepared from lemons obtained from a local supermarket in Hanoi, Vietnam. The chemicals were used as received and their solutions were prepared in double distilled water.

Fluorescence measurements were carried out using a Jasco FP-6500 spectrofluorometer (Japan). Absorption spectra were collected on an Agilent 8453 UV-Vis spectrophotometer (USA). Fourier Transform Infrared (FTIR) spectra were recorded by a Nicolet iS50 spectrometer from Thermo Scientific (Japan). Morphologies and sizes of the products were characterized using a JEOL JEM-1010 Transmission Electron Microscope (TEM), and a JEOL JSM-IT800 Field Emission Scanning Electron Microscope (FE-SEM) (Japan). The elemental analysis was recorded on the Oxford EDX Ultim Max 65 (England) coupled with the FE-SEM instrument. Raman spectra of the samples were taken with acquisition time of 60 s in 2 cycles by using HR 800 spectrometer from Horiba Jobin Yvon (France). The size distribution was characterized using a Dynamic Light Scattering (DLS) Instrument (Horiba SZ-100-Z2, Japan). Glass chromatographic column with 45 cm in length and 2 cm in diameter purchased from Duran, Germany.

### 1.2 Preparation of Au@NCDs composite

The synthesis conditions of Au@NCDs composites were primarily optimized including NCDs:$HAuCl_4$ ratio, temperature and reaction time (Table S1). The Au@NCDs composite was obtained by adding 2.5 mL of a NCDs solution to 2.5mL $HAuCl_4$ soulution under continuous stirring within 5 minutes and heating. As a result, a composite with a pink color (Figure S2) formed by a ratio of 2.5 mL NCDs (0.15 mg/L) and $HAuCl_4$ (0.2 mM) after 5 minute at 60$^o$C exhibited the most intense fluorescence intensity, which was choosen for further studies.

**Table S1:** Synthesis conditions of Au@NCDs composites.

| Vials' labels | NCDs (mg/L) | $HAuCl_4$ (mM) | Temperature |
|---|---|---|---|
| 5' | 0.15 | 0.2 | 70°C |
| 1 | 0.15 | 0.25 | 60°C |
| 2 | 0.10 | | |
| 3 | 0.075 | | |
| 4 | 0.06 | | |
| 5 | 0.15 | 0.2 | |
| 6 | 0.10 | | |
| 7 | 0.075 | | |
| 8 | 0.06 | | |
| 9 | 0.15 | 0.15 | |
| 10 | 0.10 | | |
| 11 | 0.075 | | |

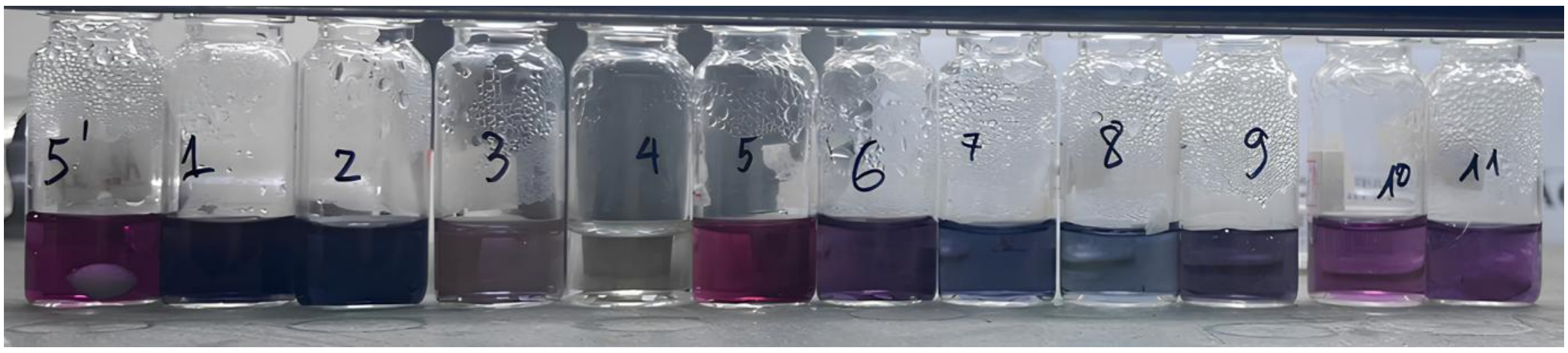


**Figure S1:** The Au@NCDs samples with with varying concentrations of NCDs and $HAuCl_4$.

## 2. Computational details

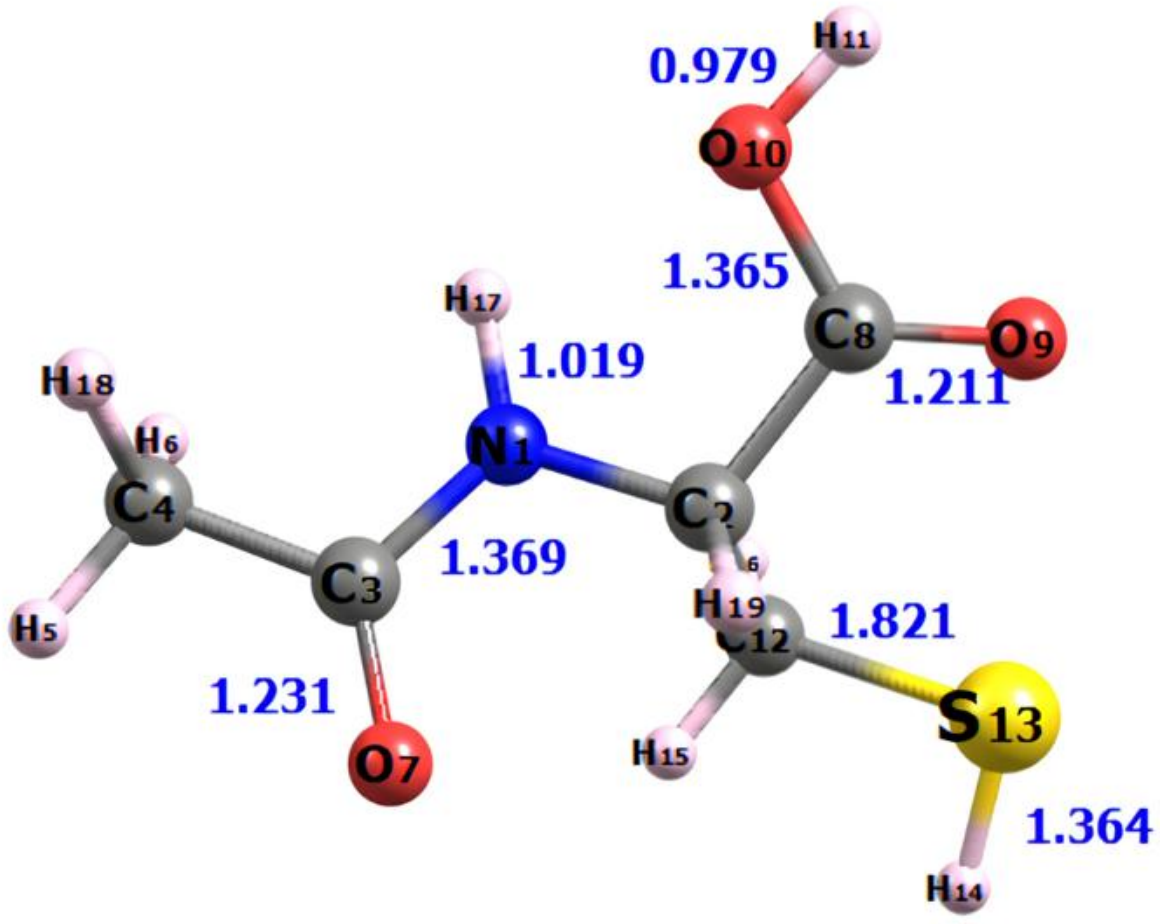


**Figure S2:** Optimized structure and atomic numbering scheme of NAC.

**Table S2:** Calculated and experimental structural parameters of NAC with corresponding deviations

| Structural parameter | This work | References [1] | Deviation,% |
|---|---|---|---|
| O10-C8, Å | 1.365 | 1.326 | 2.94 |
| C8-O9, Å | 1.211 | 1.191 | 1.68 |
| C3-O7, Å | 1.231 | 1.245 | 1.12 |
| C3-N1, Å | 1.369 | 1.324 | 3.40 |
| <C4C3N1C2, degree | 175.860 | 177.95 | 1.17 |
| <O7C3N1, degree | 120.89 | 122.83 | 1.58 |
| <N1C2C8, degree | 109.30 | 107.37 | 1.80 |

## 3. Photophysical measurements

### *Raman spectroscopy*

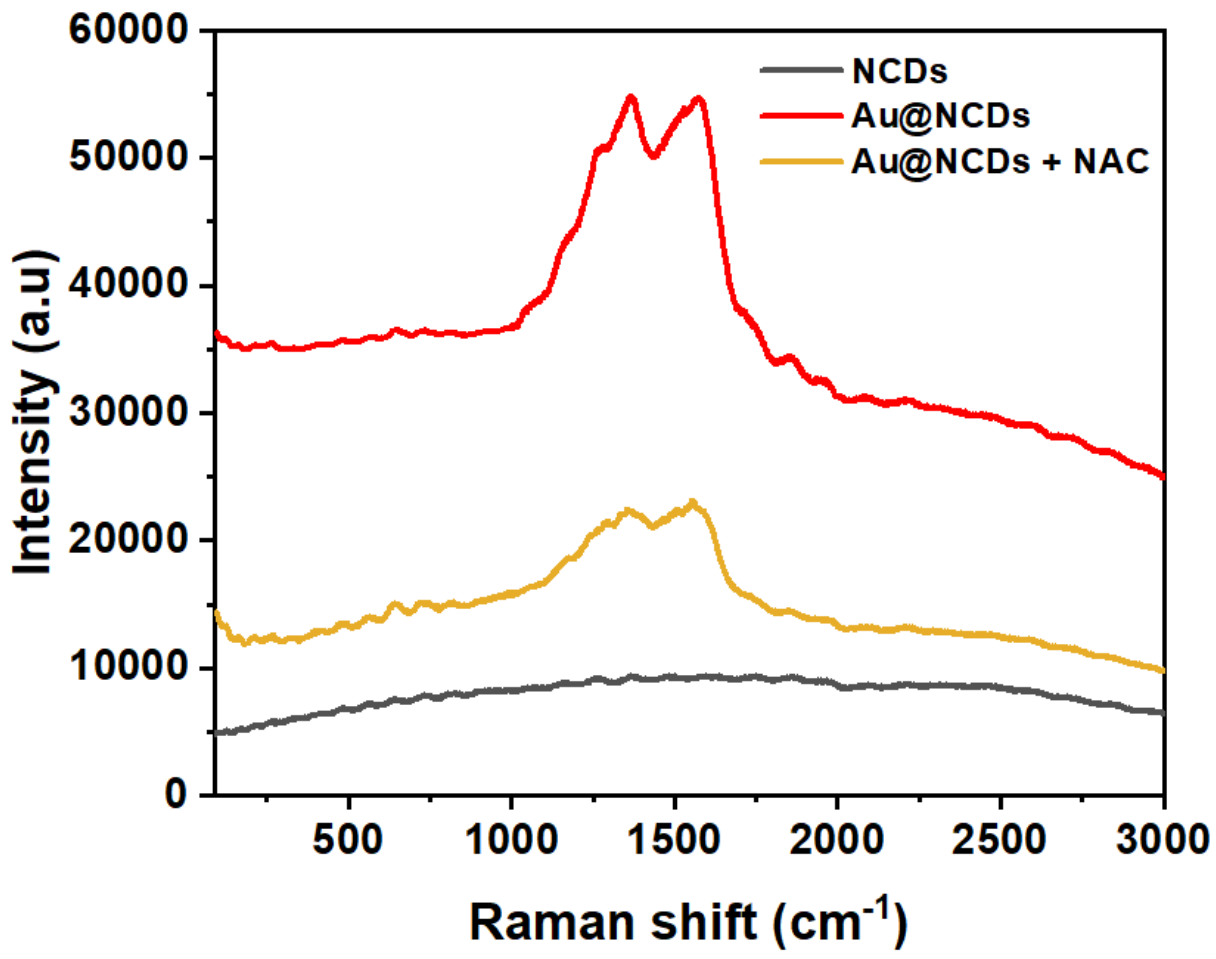


**Figure S3:** Raman spectra of NCDs, Au@NCDs and Au@NCDs-NAC.

### *Excitation and emission spectroscopy of NCDs*

The optical properties of the NCDs are clearly demonstrated in Figure S3. The excitation spectrum (Fig. S3, left) reveals a strong absorption band in the range of 300–380 nm with a maximum at approximately 355 nm, indicating an efficient light-harvesting capability in the UV region. The photoluminescence spectrum (Fig. S3, right), recorded at an excitation wavelength of $\lambda_{ex}$ = 355 nm, exhibits an intense emission peak centered around 442 nm, confirming the blue fluorescence emission of the NCDs.

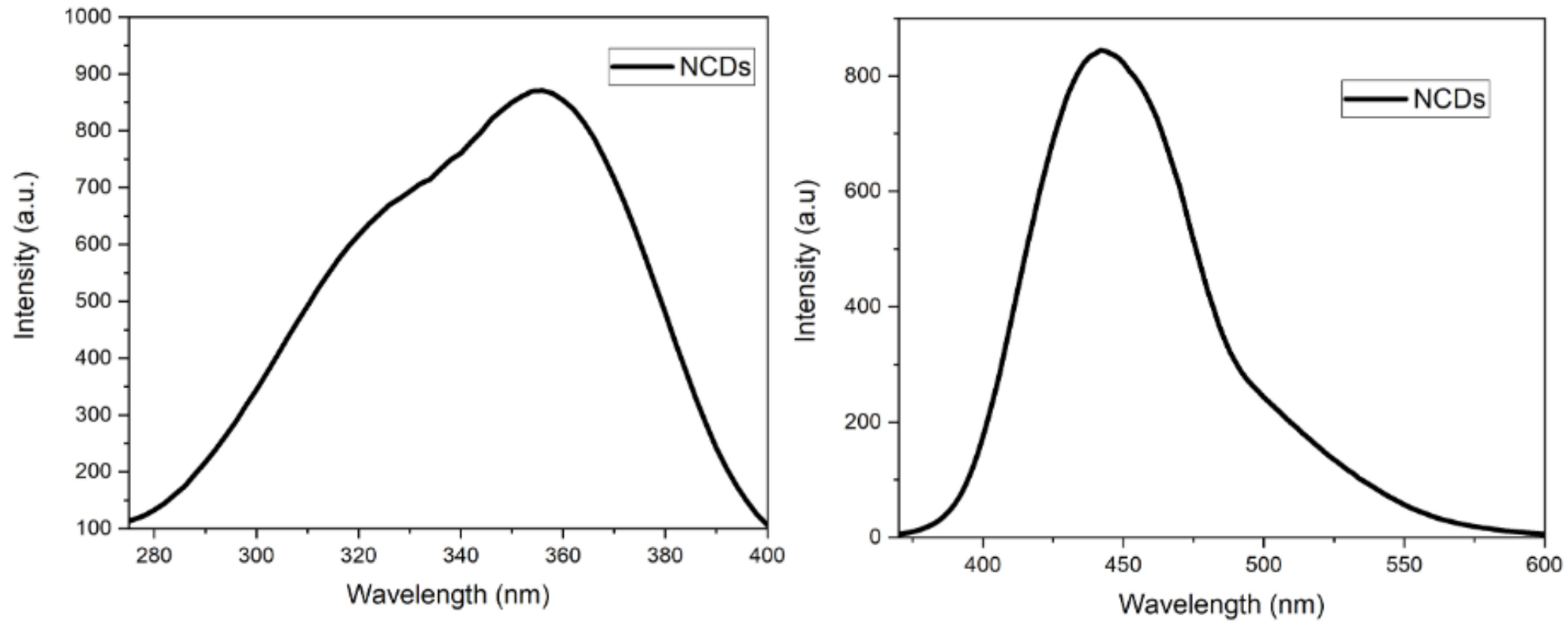


**Figure S4:** (left) Excitation spectra of NCDs (0.15 mg/mL), $\lambda_{ob}$ = 442 nm; (right) Photoluminescent spectra of NCDs (0.15 mg/mL) at $\lambda_{ex}$ = 355 nm.

### ***Fluorescent response of Au@NCDs probe with time***

The fluorescence intensity of Au@NCDs at 446 nm in a 25 mg/L NAC solution exhibited a slight decrease over time. To allow for stabilization and ensure reliable measurements, all subsequent readings in this study were taken 5 minutes after each experimental manipulation.

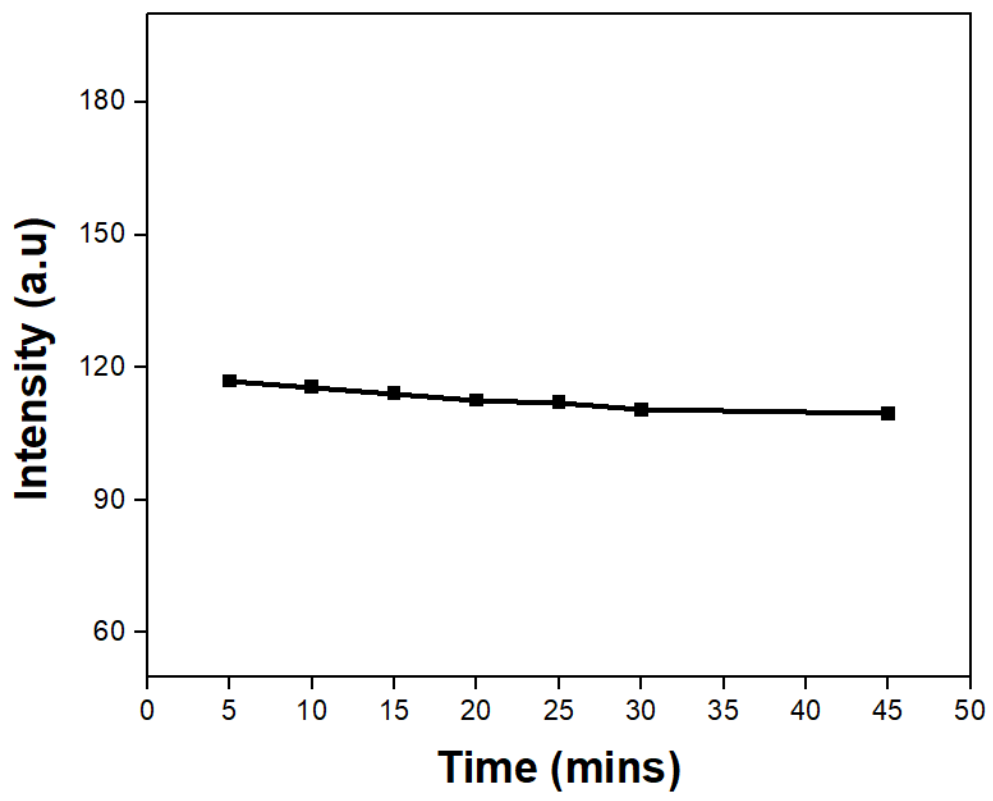


**Figure S5:** Fluorescent response of Au@NCDs probe with time.

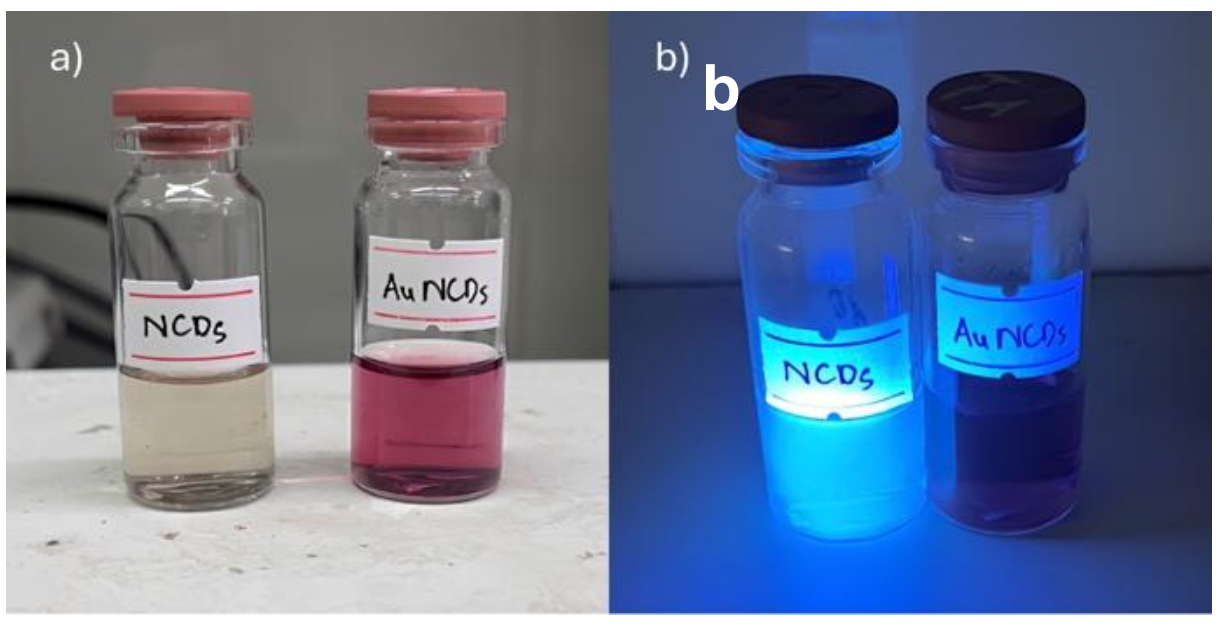


**Figure S6:** Photographs of NCDs and Au@NCDs under a) day light and b) 354 nm UV light.

***Determination of Citric Acid Concentration by Acid–Base Titration***

*1. Titration Procedure:*

About 0.5mL of lemon juice was transferred into a conical flask and slightly diluted with pure water. Phenolphthalein (7–8 drops) was added as an indicator. The sample was titrated with NaOH 0.1M solution until a persistent faint pink color was observed. The consumed NaOH volume at the endpoint was recorded. Measurements were performed in triplicate.

*2. Calculation of citric acid concentration:*

Citric acid ($C_6H_8O_7$) is a triprotic acid and reacts with NaOH according to the following stoichiometric equation:

$$C_6H_8O_7 + 3NaOH \rightarrow Na_3C_6H_5O_7 + 3H_2O$$

The concentration of citric acid was calculated using:

$$C_{citric} = \frac{C_{NaOH} \times V_{NaOH}}{3 \times V_{sample}}$$

where $C_{\mathrm{NaOH}}$ is the molar concentration of NaOH (0.100 M),

$V_{\mathrm{NaOH}}$ is the volume of NaOH at the equivalence point (5.50 mL), and

$V_{\mathrm{sample}}$ is the sample volume (0.50 mL).

Substituting the experimental values (volumes expressed in liters):

$$C_{\mathrm{citric}} = \frac{0.1 \times 5.5 \times 10^{-3}}{3 \times 0.5 \times 10^{-3}} = 0.37\ \mathrm{M}$$

The citric acid concentration was calculated to be 0.37 M.

**4. Analytical performance comparison**

Fluorescent sensors based on N,S,Co-CQDs, Poly(thymine)-templated CuNPs, and ratiometric chiral CDs can achieve nanomolar detection limits with 0.011 mg/L and 0.0082 mg/L, demonstrating high analytical sensitivity. In contrast, colorimetric AuNPs and fluorescent PEI-CDs sensors offer simple, rapid (2–5 min), and cost-effective detection, while DNA templates and biocompatible metal ions (e.g., $Fe^{3+}$ and $Mn^{2+}$) enhance biosafety. Notably, smartphone-integrated ratiometric chiral CDs enable accurate on-site analysis with HPLC-comparable performance (Table S3). Despite their excellent photostability, biocompatibility, and physicochemical stability, these sensing platforms still face several limitations. Their selectivity can be compromised by interference from structurally similar thiol-containing amino acids, particularly cysteine and homocysteine. Furthermore, fluorescence signals may gradually deteriorate over time due to natural oxidation, while sensing performance is highly dependent on the pH of the analytical medium. Therefore, careful control of these factors is essential to ensure the accuracy, reliability, and practical applicability of these sensors in the analysis of complex biological and pharmaceutical samples.

**Table S3:** Analytical performance comparison.

| No. | Sensor | Linear range (mg/L) | Detection limit (mg/L) | Sensing mechanism | Ref. |
|---|---|---|---|---|---|
| 1 | AuNCDs | 4.0 – 20.0 | 0.863 | Binding affinity via Au–S interactions | This work |
| 2 | AuNPs: colorimetric probe | 0,49 −16,30 | 0,44 | Inhibition of in situ gold nanoparticle (AuNP) formation | [2] |
| 3 | N, S, Co-Doped Carbon Quantum Dots | range 1: 0,11 – 4,17 range 2: 4,17 – 31,59 | 0,011 | "Turn-on" fluorescence recovery of the N, S-CQDs-$Fe^{3+}$ system via a redox reaction, where the sulfhydryl group of NAC reduces $Fe^{3+}$ to $Fe^{2+}$ | [3] |
| 4 | Poly(thymine)-templated fluorescent copper nanoparticles | 0,033 – 16,32 | 0,0082 | NAC forms Cu–S coordination bonds with copper nanoparticles. | [4] |
| 5 | Branched polyethylenimine-functionalized carbon dots: fluorescent probes | 0,91 – 45,34 | 0,091 | Reduction of $Cu^{2+}$ to $Cu^{+}$ by NAC inhibiting electron transfer and restoring PEI-CD fluorescence. | [5] |
| 6 | Portable ratiometric fluorescent sensor based on chiral carbon dots | 1,63 – 19,58 | 0,0071 | Static quenching: NAC disrupts the D-NCDs–L-Arg complex via strong nucleophilic interaction. | [6] |
| 7 | Metal ion-mediated carbon dot nanoprobe | 6,53 – 816,0 | 4,90 | The competitive coordination of NAC with $Mn^{2+}$ ions | [7] |

## 5. References


(1) Wu, Q.-A.; Xu, Z.-Y. *Acta Crystallogr. Sect. E Struct. Rep. Online* **2004**, *60*, o949–o950. doi:10.1107/S1600536804010505

(2) Rastegarzadeh, S.; Hashemi, F. *Analytical Methods* **2015**, *7*, 1478–1483. doi:10.1039/C4AY01961J

(3) Li, Y.; Hu, Y.; Jia, Y.; Jiang, X.; Cheng, Z. *Anal. Lett.* **2019**, *52*, 1711–1731. doi:10.1080/00032719.2019.1566349

(4) Wang, H.-B.; Zhang, H.-D.; Chen, Y.; Ou, L.-J.; Liu, Y.-M. *Analytical Methods* **2015**, *7*, 6372–6377. doi:10.1039/C5AY00841G

(5) Liu, H.; Li, R. S.; Zhou, J.; Huang, C. Z. *Analyst* **2017**, *142*, 4221–4227. doi:10.1039/C7AN01136A

(6) Wei, S.; Hu, K.; Yan, Y.; Wang, F.; Bai, B.; An, Y.; Sun, G. *Spectrochim. Acta A Mol. Biomol. Spectrosc.* **2026**, *362*, 128206. doi:10.1016/j.saa.2026.128206

(7) Wang, Q.; Zhu, B.; Han, Y.; Yang, X.; Xu, Y.; Cheng, Y.; Liu, T.; Wu, J.; Li, S.; Ding, L.; Bai, J.; Niu, Y. *Luminescence* **2022**, *37*, 1267–1274. doi:10.1002/bio.4292
